# Impact of the COVID-19 pandemic on cardiology fellow echocardiography education at a large academic center

**DOI:** 10.1186/s12909-022-03880-z

**Published:** 2022-12-13

**Authors:** Merilyn S. Varghese, Jordan B. Strom, Joseph P. Kannam, Sarah E. Fostello, Marilyn F. Riley, Warren J. Manning

**Affiliations:** 1 Department of Medicine (Cardiovascular Diseases), Beth Israel Deaconess Medical Center, Harvard Medical School, 330 Brookline Avenue, 02215 Boston, MA USA; 2 Richard A. and Susan F. Smith Center for Outcomes Research in Cardiology, Boston, MA USA; 3 Non-Invasive Cardiology Department, Beth Israel Lahey Health- Plymouth Hospital, Plymouth, MA USA; 4 Department of Radiology, Beth Israel Deaconess Medical Center, Harvard Medical School, Boston, MA USA

**Keywords:** Echocardiography, COVID-19, Fellowship, Education

## Abstract

**Background:**

In response to COVID-19 pandemic state restrictions, our institution deferred elective procedures from 3/15/2020 to 6/13/2020, and removed cardiology fellows from the echocardiography rotation to staff clinical services. We assessed the impact of the COVID-19 pandemic on fellow education and echocardiography volumes.

**Methods:**

Our institutional database was used to examine volumes of transthoracic (TTE), stress (SE), and transesophageal echocardiograms (TEE) from 7/1/2018 to 10/10/2020. Study volumes were compared in three intervals: pre-pandemic (7/1/2018- 3/14/2020), pandemic (3/15/2020–6/13/2020), and pandemic recovery (6/14/2020–10/10/2020). We examined weekly number of TTEs performed or interpreted by cardiology fellows during the study period, and compared these to the two previous academic years.

**Results:**

Weekly TTE volume declined by 54% during the pandemic, and increased by 99% during pandemic recovery, (*p* < 0.05). SE and TEE revealed similar trends. A strong correlation between weekly TTE volume and inpatient admissions was observed during the study period (r_s_=0.67, *p* < 0.05). Weekly fellow TTE scans declined by 78% during the pandemic, with a 380% increase during pandemic recovery (*p* < 0.05). Weekly fellow TTE interpretations declined by 56% during the pandemic, with a 76% increase during pandemic recovery (*p* < 0.05).

**Conclusion:**

COVID restrictions between 3/15/2020- 6/14/2020 coincided with a marked decline in TTE, SE, and TEE volumes, with an increase similar to near pre-pandemic volumes during the pandemic recovery period. A similar decline with the onset of COVID restrictions, and increase to pre-restriction volumes thereafter was observed with fellow scans and interpretations, but total academic year fellow training volumes remained depressed. With the ongoing COVID-19 pandemic and rise of multiple variants, training programs may need to adjust fellows’ clinical responsibilities so as to support achievement of echocardiography training certification.

## Background

The impact of the COVID-19 pandemic on echocardiography volume and fellow echocardiography education has not been fully assessed. Many echocardiography labs met the declaration of COVID-19-related state-wide emergencies by deferring non-urgent studies [1–3]. Marked declines in TTE volume were observed in March 2020 at an academic center after the implementation of a new protocol to comply with state mandates [2]. In Massachusetts, a state of emergency was declared on March 10, 2020 [4, 5], and our institution deferred non-urgent echocardiographic procedures from March 15, 2020 to June 13, 2020. We evaluated the study period from July 2018 to October 2020 to understand the impact of the COVID-19 pandemic and the deferment of non-urgent procedures on echocardiography volume.

Echocardiography training requires familiarity with scanning and interpreting echocardiograms. Board certification, a surrogate for competency in echocardiography and independent practice, is granted based on five factors: (a) a valid medical license, (b) board certification in internal medicine, cardiology, or anesthesiology, (c) training in an adult cardiovascular disease program, (d) passing of the National Board of Echocardiography examination, and (e) scan and interpretation numbers [6]. At the time of this study, the American Society of Echocardiography recommends 150 transthoracic echo (TTE) scans and 300 interpretations for level II certification [7]. Fellows in programs throughout the United States demonstrate their competency by passing the echocardiography board exam and completing the requisite number of scans and interpretations.

Cardiology fellowship programs responded to the onset of the pandemic in March 2020 in varied ways; 38% of fellows nationally reported being reassigned to cover non-cardiology services, and 88% of fellows reported decreased fellow staffing in echocardiography laboratories [8]. Our fellowship program, similarly, removed cardiology fellows from the echocardiography rotation to cover non-cardiology services, and to serve as back-ups for their colleagues. Fellows continued to perform on-call emergency echocardiograms on evenings and weekends. An online virtual echocardiography teaching session was enacted in April 2020 to promote education and address concerns about echocardiography training. We evaluated the impact of COVID-19 on fellow scans and interpretations to guide clinicians and educators during possible future pandemics, and the current COVID-19 pandemic with variants on the rise [9].

## Methods

### Study design and data source

A cross-sectional study design was used to perform a retrospective review of the TTE, stress echo (SE) and transesophageal echo (TEE) volume from 07/1/2018 to 10/10/2020 [10, 11]. Data were derived from our institutional echocardiography database. This study was deemed to be institutional review board (IRB) exempt.

### Setting and participants

We compared study volumes for all studies performed at our institution in three intervals: pre-pandemic (7/1/2018- 3/14/2020), pandemic (3/15/2020–6/13/2020) and pandemic recovery (6/14/2020–10/10/2020). For outpatient and inpatient TTEs, the pre-pandemic period was 5/26/2019-3/14/2020. We also examined all weekly TTE scans performed or interpreted by first-year and second-year general cardiology fellows in the pre-pandemic, pandemic, and post-pandemic intervals.

### Statistical methods

One-way analysis of variance (ANOVA) testing compared the effect of these intervals on study volume, with post-hoc comparisons using Bonferroni correction. ANOVA assumes normality, independence, and equal variances, or homoskedasticity, between groups. Student’s t-test was used to compare the average percent difference between pandemic and pandemic recovery volume for inpatient and outpatient TTE. Spearman’s correlation coefficient, which can be used to compare two continuous variables without normal distributions, was computed to determine whether there was a relationship between studies performed and admissions. Scans and interpretations for fellows were compared for the 2017 to 2020 academic years, with academic years starting on July 1st, using ANOVA. Data were analyzed using Stata Version 17 (Stata Corporation, TX, USA) with a 2-tailed *p*-value of < 0.05 to denote significance.

## Results

### Echocardiography volume

**Fig. 1:**
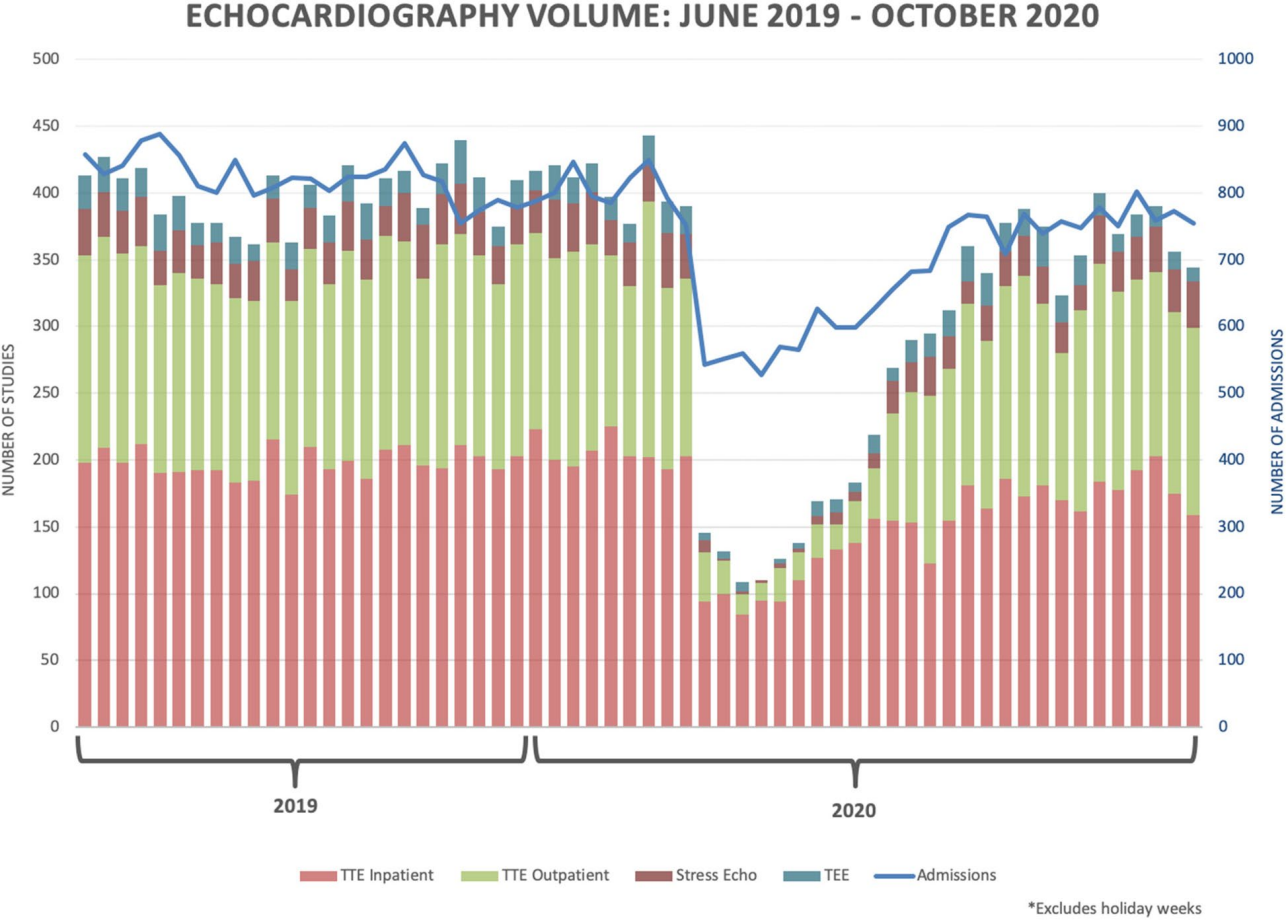
Weekly echocardiographic volume for transthoracic echo (TTE), stress echo (SE) and transesophageal echo (TEE). A steep decline is seen with the onset of pandemic restrictions, corresponding to March 15, 2020, with a gradual increase is observed during pandemic recovery. Hospital admissions are also shown and follow a similar trend, as denoted by the blue line

A total of 42,375 studies were performed from July 1, 2018 to October 10, 2020. When comparing the weekly pre-pandemic (337 ± 24, mean ± SD) to the weekly pandemic period (156 ± 49), a 54% decline was observed (*p* < 0.05). When comparing pandemic to pandemic recovery (311 ± 29), there was a 99% weekly average increase (*p* < 0.05, Fig. 1). For the pre-pandemic to the pandemic period, there was a greater decline in outpatient than inpatient TTEs: -75 ± 18% versus − 40 ± 13% (*p* < 0.05). For pandemic to pandemic recovery, there was a decline of -7 ± 10.5% versus − 14 ± 9% for outpatient vs. inpatient TTEs, which was not significant (*p* = 0.08). Weekly TTE volumes were strongly correlated with the number of inpatient admissions throughout the study period (r_s_=0.67, *p* < 0.05).

For SE, the weekly pre-pandemic volume of (30 ± 6) declined to (8 ± 8) during the pandemic (*p* < 0.05), and rose during pandemic recovery (28 ± 6, *p* < 0.05). For TEE, there was a significant weekly decline from pre-pandemic to pandemic (21 ± 5 to 8 ± 5, *p* < 0.05), and increase post pandemic (19 ± 5, *p* < 0.05).

### Fellow echocardiography involvement

**Fig. 2:**
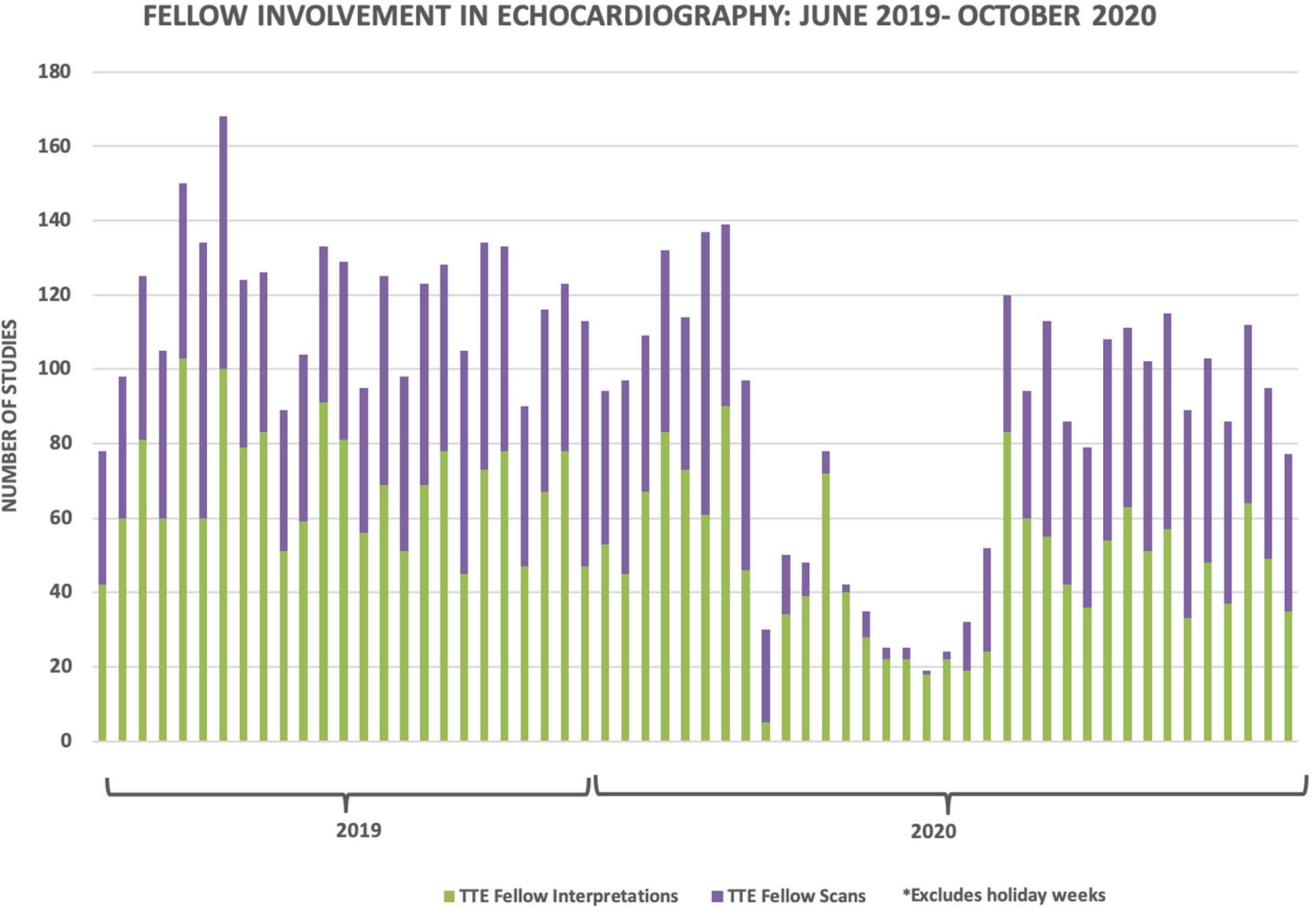
Weekly echocardiographic fellow scan and interpretive volume for transthoracic echo (TTE). Similar to Fig. 1, a sharp decline is seen in fellow echocardiography involvement with the onset of pandemic restrictions. During pandemic recovery, a similar increase is again seen

For fellow TTE scans pre-pandemic to pandemic, there was a 78% weekly decline (46 ± 9 to 10 ± 9, *p* < 0.05), with a 380% weekly increase during recovery (48 ± 7, *p* < 0.05). Similarly, there was a 56% decline in fellow weekly TTE interpretations during the pandemic (66 ± 19 to 29 ± 17), which increased by 76% during recovery (51 ± 14, *p* < 0.05, Fig. 2).

Fellows during the 2019–2020 year had lower numbers of TTE scans and interpretation volumes compared to the prior two academic years. Average weekly number of scans per fellow declined in 2019–2020: 92 ± 45, compared to 2017–2018: 112 ± 38, and 2018–2019: 115 ± 48 (*p* = 0.32). Average total number of interpretations also declined per fellow for 2019–2020: 196 ± 54, compared to 2017–2018: 225 ± 59 and 2018–2019: 266 ± 127 (*p* = 0.09).

## Discussion

This cross-sectional study highlights the effect of reduced study volume and removal of fellows from echocardiography rotations. This study had three main findings. First, echocardiography volumes for TTE, SE, and TEE markedly declined with the onset of COVID restrictions and increased during pandemic recovery to near pre-pandemic levels. Second, TTE volumes strongly correlated with inpatient admissions throughout the study period. Third, though weekly fellow TTE scan and interpretations increased during the pandemic recovery period to near pre-pandemic levels, fellows in the 2019–2020 academic year had lower total scan and interpretation volumes than fellows in previous academic years. These numbers are likely explained by the dual impact of removing fellows from the echocardiography rotation and the reduction in overall echocardiography volumes during the pandemic period. Fellows in the 2019–2020 academic year risked not meeting volumes for level II TTE certification, a marked disparity compared to the past three decades at our institution [7].

Prior reports evaluating the impact of the COVID-19 pandemic on echocardiography volume and education are limited. Declines in echocardiography volume were observed with the onset of the pandemic in March 2020 at an academic center in Chicago [2]. Between March 2020 and May 2020, declines were observed in TTE, SE, and TEE volume at an academic center in New York City [12]. To our knowledge, our study is the first to report on the recovery of TTE, SE, and TEE volumes after the onset of the COVID-19 pandemic, and quantify the impact on fellow echocardiography volumes.

Due to concerns raised by fellowship programs regarding the impact of the COVID-19 pandemic, the National Board of Echocardiography extended the time required to obtain procedural experience by one year for trainees affected by the pandemic [13]. Fellowship programs have had to explore alternative options to increase fellow echocardiography exposure— including virtual reading sessions with attending physicians, online lectures, and hands-on learning with simulation software [3, 14]. In our center, the implementation of virtual reading sessions increased fellow interpretation numbers. Future studies should compare the impact of these teaching modalities on overall scan and interpretation numbers, and knowledge retention.

### Limitations

Our study had limitations as data were derived from a single academic medical center without a control group to account for secular trends. Similar declines after the onset of the COVID-19 pandemic were seen in Chicago and New York City [2, 12]. Improvement in these numbers in the pandemic recovery period should be confirmed with other centers.

## Conclusion

During the first wave of the COVID-19 pandemic, removing fellows from echocardiography rotations to cover inpatient responsibilities and to ensure a backup pool of fellows risked indispensable echocardiography training. In preparation for future waves of the pandemic, alternative echocardiography teaching modalities and backup strategies should be evaluated.

## Data Availability

The datasets used and/or analyzed during the current study are available from the corresponding author on reasonable request.

## Abbreviations

COVID-19: Coronavirus disease
IRB: Institutional Review Board
SE: stress echocardiogram
TEE: transesophageal echocardiogram
TTE: transthoracic echocardiogram
